# Chinese herbs and their active ingredients for activating *xue* (*blood*) promote the proliferation and differentiation of neural stem cells and mesenchymal stem cells

**DOI:** 10.1186/1749-8546-9-13

**Published:** 2014-04-09

**Authors:** Yin-chu Si, Qiang Li, Chun-e Xie, Xin Niu, Xiao-hui Xia, Chang-yuan Yu

**Affiliations:** 1 Department of Anatomy, Beijing University of Chinese Medicine, Chaoyang District, Beijing 100029, China; 2 Department of Phytochemistry, School of Chinese Materia Medica, Beijing University of Chinese Medicine, Chaoyang District, Beijing 100029, China; 3 Department of Gastroenterology, Dongfang Hospital, Fengtai District, Beijing, China; 4 Department of Physiology, Beijing University of Chinese Medicine, Chaoyang District, Beijing, China; 5 Insititute of China Resources Double-crane Pharmaceutical Co., Ltd. Chaoyang District, Beijing, China; 6 College of Life Science and Technology, Beijing University of Chemical and Technology, Chaoyang District, Beijing 100029 Beijing, China

## Abstract

Some Chinese herbs are anti-thrombolysis, and anti-inflammatory, improves brain RNA content, promotes brain protein synthesis, enhances dopamine function, regulates brain hormones, and improves microcirculation in central nervous system that might improve, repair and rehabilitation from the stroke and brain injury. Specific Chinese herbs and their components, such as *Acanthopanax, Angelica,* could maintain the survival of neural stem cells, and *Rhodiola*, *Ganoderma spore Polygala, Tetramethylpyrazine*, *Gardenia, Astragaloside and Ginsenoside Rg1* promoted proliferation of neural stem cells, and *Rhodiola, Astragaloside* promoted differentiation of neural stem cell into neuron and glia *in vivo. Astragalus, Safflower, Musk, Baicalin, Geniposide, Ginkgolide B, Cili polysaccharide, Salidroside, Astragaloside, Antler polypeptides, Ginsenoside Rg1, Panax notoginseng saponins* promoted proliferation and differentiation of neural stem cells *in vitro. Salvia, Astragalus, Ginsenoside Rg1, P. notoginseng saponins, Musk polypeptide, Muscone and Ginkgolide B* promoted neural-directed differentiation of MSCs into nerve cells. These findings are encouraging further research into the Chinese herbs for developing drugs in treating patients of stroke and brain injury.

## Introduction

Neural stem cells (NSC) in the central nervous system are capable of self-replication, proliferation, migration and differentiation [1]. They can be differentiated into the essential cells for the brain and spinal cord tissue, including neurons, astrocytes and oligodendrocytes [2]. This discovery contravened the dogma [3] that new neurons involving in the repair of nerve tissue cannot be produced after neuronal degeneration and necrosis in the central nervous system (CNS). Therapy using NSC technology has emerged as a viable alternative treatment for neural injury [4].

There are two intervention strategies involving NSCs in the treatment of CNS injury and degenerative diseases. The first strategy involves endogenous NSCs to repair the lesion sites [5], but it is an issue that the proliferation of endogenous NSCs is insufficient [6]. Methods including chemotherapy, stimulation of endogenous NSC proliferation, and induced directional migration and differentiation are under research [7]. The second strategy involves the exogenous transplantation of NSCs into the lesion sites [8]. Transplanted NSCs must survive, replicate, and differentiate into local nerve cells, for repairing the injury [9]. The requirements of precursor cells or nerve cells for directional induction at the lesion sites have been under research. The efficacy of Chinese medicine (CM) in the prevention and treatment of diseases of the central nervous system has been a major research issue for decades in the neuroscience and medicine [10].

After screening a number of Chinese herbs and their active ingredients *in vitro* and *in vivo*, CM was demonstrated to have various effects on NSCs in many aspects [11]. This study aims to focus on the efficacy and roles of CM in the survival, self-proliferation, directed migration, and differentiation of NSCs.

### Methods for studying proliferation and directed differentiation of neural stem cells *in vivo*

Endogenous NSCs are restricted to two main regions: subgranular zone (SGZ) of the hippocampal dentate gyrus (DG) and the subventricular zones (SVZ) of the lateral ventricles in adults [12,13]. Cells in the SVZ proliferate and form the rostral migratory stream to the olfactory bulb, and then differentiate into granular cells [14]. Cells in the SGZ proliferate and migrate to the granular cell layer, and then differentiate into new granule cells [15]. There are also self-replicating NSCs in other parts of brain, such as the cortex and striatum, which can differentiate into cerebral nerve cells [16]. In the case of injury activated, NSCs proliferate and migrate to the sites of injury, differentiate into new nerve cells, replace damaged nerve cells, participate in the formation of new neural circuits, and promote structural and functional repair [17,18].

The number of neural cells induced by brain damage stimulation on SVZ NSC proliferation is too limited to repair the injury [6]. Some Chinese herbs (*e.g.*, *Panax ginseng*, *Panax notoginseng*) improve rehabilitation from brain injury and degenerative diseases [10]. The Chinese herbs and their active ingredients (*e.g.*, ginsenosides) provide a promote the proliferation and differentiation of NSCs [19].

### Animal models and screening methods *in vivo*

Rodent models of transient global cerebral ischemia, hypotonic hypoxia (which causes embryonic, intrauterine hypoxia), spinal cord injury, cerebral ischemia, chronic stress, Alzheimer’s disease (AD), middle cerebral artery occlusion, cerebral ischemia, and cerebral hemorrhage are available for screening Chinese herbs [20]. For oral administration, a single herb decoction (*e.g.*, *Acanthopanax, Rhodiola.*) was usually employed. For intravenous and intraperitoneal injections, active components (*e.g.*, *Astragaloside ginsenosides* Rg1*)* of Chinese herbs were often used. The numbers of nestin- and BrdU-positive cells in Chinese herb treatment groups (*Acanthopanax, Rhodiola and Astragaloside ginsenosides* Rg1) were statistically different from the control groups (*P* < 0.05 or 0.01) in stroke rat brain [20,21], indicating significant effects of the treatments on the self-replication and proliferation of NSCs. The numbers of cells double-positive for β-tubulin III/BrdU, olig2/BrdU and GFAP/BrdU were also changed by the treatment with Chinese herbs and their components, indicating their effect on the differentiation of NSCs [21]. The effects of Chinese medicines and active ingredients on the proliferation and differentiation of NSCs in the SVZ, SGZ, cortex, hippocampus, striatum, and foci of ischemia, hemorrhage, have been investigated as described in the following sections.

### Effects on proliferation and differentiation *in vivo*

Table 1 summarizes the effect of Chinese herbs on differentiation of NSCs *in vivo. Acanthopanax* (*Ciwujia*) consists of dried roots and rhizomes or stems of *Araliaceae Acanthopanax*[22]. *Ciwujia* increased nestin-positive cell number in Sprague–Dawley rats with cerebral ischemic lesions 5–7 days after transient global cerebral ischemia with rat tail vein injection. These findings were consistent with the improvement of neurological functions [23]. *Angelica* (*Danggui*) is the dried roots of *Angelica Umbelliferae*[24], which increased the number of nestin-positive cells in intrauterine hypoxia brain after angelica injection (250 g/L *Angelica* injection, 8 mL/kg body weight) [25]. *Rhodiola* (*Hongjingtian*) is the dried roots or rhizomes of sedum *Rhodiola crenulata*[26]. *Hongjingtian* decoction (1.5 g/kg) increased the percentages of BrdU-labeled cells and BrdU/β-tubulin III double-labeled cells in the SGZ of the hippocampal dentate gyrus after for 12 days of Oral administration to depressive rats [27]. *Rhodiola rosea* (15 mg/kg) increased the number of BrdU-positive cells, the percentages of BrdU- and β-tubulin III double-labeled cells and the numbers of neurons in the hippocampus of depressive rats [27]. *Ganoderma* (*lingzhi*) is the dried fruiting bodies of the Polyporaceae fungus *Ganoderma lucidum*[26]. *Ganoderma* spore solution (8 g/kg/d) increased BrdU-positive staining quantity in central canal ependymal cells of the rat spinal cord after gastric feeding (2 mL) twice a day after T12 spinal cord injury [28]. Some BrdU-positive cells in the spinal cord white matter simultaneously expressed oligedendroyte-specific proteins, nestin, neurofilament proteins (NF) or glial fibrillary acidic protein (GFAP). *Polygala* (*Yuanzhi*) is the dried roots of *Polygalaceae*[29], and increased the numbers of BrdU-positive cells in the DG of the hippocampus in the AD mouse [30]. The numbers of BrdU-positive cells in the DG were positively correlated with spatial memory scores in each group of mice with different doses of *Polygala* (r = 0.624; *P* < 0 01).

**Table 1:** **The role of Chinese herbs and its active ingredients on proliferation and differentiation of neural stem cells *in vivo***

**Chinese herbs and active ingredients**	**Animal model**	**Sites of neural stem cells**	**The role of proliferation**	**The role of differntiation**
*Acanthopanax*[22,23]	Transient global cerebral ischemia in rats	Hippocampus, ependymal, subependymal region, ischemic area	Nestin positive cells↑	
*Angelica*[24,25]	The intrauterine hypoxia embryonic rats	Whole brain	Nestin positive cells↑	
*Rhodiola*[26,27]	Chronic stress rats	Hippocampus	BrdU positive cells↑	BrdU/β-tubulin ↑. Neuronal directed differentiation
Ganoderma spore [26,28]	Spinal cord injury in rats	Central canal ependymal zone	BrdU positive cells↑	BrdU/NF; BrdU/GFAP; BrdU/Oligo↑
*Polygala*[29,31]	Mouse AD model (D-galactose + sodium nitrite intraperitoneal injection)	SGZ	BrdU positive cells↑	
Tetramethylpyrazine [32]	Ischemia-reperfusion of cerebral ischemia in rats	SGZ	BrdU positive cells↑	
*Gardenia*[33]	Chronically stressed mice	SGZ	BrdU positive cells↑	
*Astragaloside*[32,34,35]	Transient forebrain ischemia in rats	CA1 of hippocampus, SGZ	BrdU positive cells↑	BrdU/MAP-2; BrdU/GFAP; ↑
Ginsenoside Rg1 [19,32,36-40]	MCAO rats	SVZ, SGZ, CA1 of hippocampus	Nestin, BrdU and nestin/BrdU positive cells↑	
*Panax notoginseng* saponins [19,36-39]	MCAO rats	SVZ, Hippocampus and cortex	Nestin positive cells↑	Nestin/GFAP↑
	Cortical devascularization rats	SVZ	Nestin positive cells↑	

Intraperitoneal injection of tetramethylpyrazine [40 mg/(kg•d)] one day after cerebral ischemia promoted BrdU expression in the SGZ. The effect peaked after 7 days, and remained observable for 21 days. The *Astragaloside ginsenoside* Rg1 increased the numbers of BrdU-positive cells and BrdU/GFAP double-positive cells in the rat CA1 hippocampal DG region in a transient brain ischemia model [32]. A reverse-transcription polymerase chain reaction analysis showed that *astragaloside* upregulated nerve growth factor (NGF) mRNA expression after 7 days. The *astragaloside* promoted the proliferation of NSCs in the hippocampus, and induced them to differentiate into astrocytes, which might be related to the increase in NGF mRNA expression [34,35]. Rg1 and *P. notoginseng* saponins promoted nestin and BrdU immune responses in the hippocampal DG, the subventricular zone, the CA1 region of the hippocampus, and the cortex (including the area showing ischemic involvement in MCAO rats), and peaked 7 days after ischemia [19,36-39]. Activation of NMDA receptors was involved in the effect of Rg1 on hippocampal precursor cells [40]. Rg1 increased the number of nestin-positive cells in the rats brains with cerebral hemorrhage, and improved their motor function [30]. Gardenia crude extract elevated the numbers of NeuN-positive neurons in the hippocampal DG after 8 weeks of treatment, and increased the surface density of BrdU-positive cells [33]. Gardenia crude extracts improved cognitive function in depression model mice, and promoted neurogenesis in the hippocampus, suggesting an antidepressant effect of the gardenia crude extract (Table 1).

### Effects on the proliferation and directed differentiation of NSCs *in vitro*

NSCs can be isolated, cultured, purified and identified from the cerebral cortex, hippocampus, striatum, and SVZ of embryonic and neonatal rats [2]. There were morphological changes of and monoclonal cell sphere formation by neural stem cells after induction with Chinese herbs and its effective active ingredients [39]. The numbers of nestin- and BrdU-positive cells were assessible by radioisotope labeling and immunohistochemistry. The numbers of immune double labeling positive cells of B-tubulin III/BrdU, MAP-2/BrdU, olig2/BrdU and GFAP/BrdU and flow cytometry may show the role of Chinese herbs and its components in directed differentiation.

Table 2 summarizes the roles of Chinese herbs in NSC differentiations in vitro. *Astragalus* (*Huang Qi*) injection (30 μL/mL) promoted the differentiation of NSCs into neurons in the E13 embryo rat [41,42]. An aqueous solution of *musk* (*She Xiang*) dispersed clusters of rat NSCs, increasing the numbers and lengths of neurites. An aqueous solution of *musk* (0.3%) promoted NSCs to differentiate into glial-like cells, while a higher concentration (3%) caused cytotoxicity [43].

**Table 2:** **The role of Chinese herbs and its active ingredients on proliferation and differentiation of neural stem cells *in vitro***

**Chinese herbs and active ingradients**	**Types of neural stem cells**	**The role of proliferation**	**The role of differentiation**	**Mechanism**
*Astragalus*[41,42]	14d cortical NSCs of embryonic rats		NF and GFAP positive cells↑	
Musk [43]	14d striatal NSCs of embryonic rats		Electrotransfer rate of pEGFP-C1↑Glial-like cell directed differentiation	
Baicalin [44]	Cortical NSCs of embryonic rats		β-tubulin and MAP-2 positive cells↑ ↑	
Geniposide [19]	Cortical NSCs of embryonic rats		β-tubulin positive cells↑	
Baicalin + astragaloside, [45] Baicalin + *P. notoginseng* saponins [38]	Cortical NSCs of embryonic rats		Expression of Tau↑ (adults neurons)	
Ginkgolide B [34,46,47]	Hippocampal NSCs of Neonatal rat, E14 SVZ NSCs of embryonic rats		NF cells (Has nothing to do with the concentration) ↑, GFAP cells (Positively correlated with the concentration) ↑	
	SVZ NSCs of adult rats		MAP-2 cells↑	
	SVZ NSCs of neonatal mice		β-tubulin and GFAP cells↑	
Cili polysaccharide [48]	14d embryonic rat striatal NSCs (glutamic acid damage)			Protection. Cell death rate↓; lactate dehydrogenase leakage rate↓
Salidroside [34]	Hippocampal NSCs of neonatal rats		NSE cells↑	
Quercetin-3-O-celery glucoside (CTN-986) [49]	Hippocampal NSCs of neonatal rats	MTT↑		
		3H-TdR intake↑		
	14d cortical NSCs of embryonic rats	NSCs↑		The expression of Hes5 and Cyclin D1↑
Antler polypeptides [50]	14d cortical NSCs of embryonic rats		NSE cells↑	
Ginsenoside Rg1 [21,40,51,52]	14d cortical NSCs of embryonic rats	BrdU cells↑		The expression of Hes1 and Mash1↑
TSPG [53]	Human embryonic NSCs	MTT↑	Directed differentiation of DA neuron	
*P. notoginseng* saponins [39,54]	E17 cortical and hippocampal NSCs of neonatal rats	Nestin and PCNA cells↑	Tuj1, NF, vimentin and GFAP cells↑	The expression of BDNF and bFGF↑

*Baicalin* promoted the survival of NSCs in embryonic rats with an increase of 42.3% determined by MTT assay [44]. *Salidroside* serum significantly promoted hippocampal NSCs to differentiate into neurons in a dose-dependent manner [34]. Different concentrations of *ginkgolide B* (20, 40, and 60 mg/L) added to DMEM/F12 medium promoted NSC differentiation into neuron-like cells when they were cultured for 7 or 14 days [34]. The relationship between differentiation and concentration was not significant, and the concentration had little effect on the percentage of oligodendrocytes. However, the percentage of astrocyte-like cells increased with the concentration of *ginkgolide B*[46,47]. Different concentrations of rose roxburghii tratt polysaccharide (RRTP) (20, 40, and 80 μmol/L) reduced the degree of injury of embryonic rat striatal neural stem cells caused by glutamate. A high concentration of RRTP (60 mg/L) significantly reduced mortality and the rate of leakage of lactate dehydrogenase from NSCs, suggesting a protective effect of RRTP against NSC damage [48]. *Quercetin-3-O-celery glucoside* had a distinct effect on the survival and proliferation of neonatal rat hippocampus neural precursor cells with MTT and DNA synthesis of isotopically labeled precursors 3H-TdR detection [49]. *Astragalus* saponin induced the differentiation of NSCs in the SVZ and cortex in embryonic mouse brains into neurons. This induction showed no concentration effect [55,56].

Brain microvascular endothelial cells, astrocytes and NSCs were co-cultured for 7 days with baicalin [45]. While baicalin increased the proportions of β-tubulin III-positive cell when NSCs were co-cultured with brain microvascular endothelial cells; however, it had no significant effect on the proportions of β-tubulin III-, MAP-2- and glial fibrillary acidic protein-positive cells when NSCs were co-cultured with astrocytes. When brain microvascular endothelial cells and astrocytes were co-cultured in baicalin medium, the proportion of MAP-2-positive cells was increased [45]. Baicalin increased the level of gene expression for platelet-derived growth factor in brain microvascular endothelial cells after culture for 48 h [45]. After 72 h of culture, baicalin raised the level of gene expression for vascular endothelial growth factor, nerve growth factor and platelet-derived growth factor in astrocytes. Baicalin induced the differentiation of NSCs into neurons when the stem cells were co-cultured with brain microvascular endothelial cells [45]. It also induced directed differentiation of NSCs into neurons when stem cells were co-cultured with brain microvascular endothelial cells and astrocytes. The effect of baicalin was related to the regulation of growth factor secretion from brain microvascular endothelial cells and astrocytes, and an improved microenvironment (Table 2) [45].

After the culture of embryonic rat brain cortex neural stem cells in different concentrations of antler polypeptides medium for 24 h, the resultant neurospheres produced many cells with extended protrusions, as well as numerous differentiated cells on the 7^th^ day [50]. Some cells with visible split-shaped nuclei were detected; some of these cells extended dendritic processes and formed a network. The difference in the levels of neuron-specific enolase (NSE) immunohistochemistry between cells cultured with antler polypeptides and the control group showed that the antler polypeptides promoted NSCs differentiation into neurons and suggested a potential treatment of nervous system diseases with velvet antler polypeptide.

Rg1 had effects on the proliferation of hippocampal neural precursor cells *in vitro* as shown by increased 3[H]-thymidine incorporation and colony formation rates [40]. Rg1 increased the number of BrdU-positive cells among rat fetal mouse NSCs, and upregulated Hes1 gene expression [51]. Rg1 promoted proliferation of embryonic rat SVZ NSCs and protected them against glutamate-induced damage, and this effect was relevant for increasing the percentage of STAT3-positive cells [21,52].

The ginsenoside saponins promoted the proliferation of human embryonic NSCs [54]. When ginsenoside saponins were combined with EGF and bFGF, the effect of proliferation was two times more potent than the combination of just EGF or bFGF. Ginsenoside saponins promoted directed differentiation of NSCs into dopaminergic neurons [53]. When ginsenoside saponins were combined with IL-1, the effect on proliferation was five times more potent than that induced by IL-1 alone [57]. Tanshinone (20, 40, and 80 μmol/L) exhibited a protective effect on rat NSCs affected by hypoxia and oxidants *in vitro*[57] (Table 2). Tanshinone had a greater protective effect on hypoxia damage than on oxidant damage at the same dose.

### Effects on the outcomes following transplantation of NSCs

Rat models of acute spinal cord injury (ASCI) were established by Allen’s method [58]. NSCs transfected with neurotrophin-3 (NT-3) were transplanted into the spinal cords of model rats [59]. Model rats were arbitrarily allocated into a model control (group A), a transplantation of NSCs transfected with NT-3 mediated by adenovirus group (group B), a NSC transplantation plus ginkgo biloba leaf extract group (group C), and a ginkgo biloba leaf extract group (group D). Two weeks after transplantation, the cortical somatosensory-evoked potential disappeared in groups B and C. Eight weeks after transplantation, the waveforms in groups B and C were restored to normal, but the latency was extended. At the same time, more NSCs expressing NT-3 were observed in the ependyma of the spinal cord. Six weeks later, nerve fiber bundles were seen in the lesion area, and there were more nerve fiber bundles in groups B and C than in groups A, D. Therapy with ginkgo biloba leaf extract combined with transplantation of nerve stem cells transfected with NT-3 promoted the repair or regeneration of neurons, improved conduction function, and increased the numbers of nerve fiber bundles in the injured spinal cord following acute spinal cord injury.

### Effects on the differentiation of mesenchymal stem cells into nerve cells

Table 3 summarizes the role of Chinese herbs and its active ingredients in differentiating bone marrow mesenchymal stem cells (MSCs) into nerve cells. Ginkgolide B induced the directed differentiation of MSCs in rats [60]. The percentages of NSE-positive neuron-like cells in the different concentrations of ginkgolide B were higher than the percentage in the control group. However, there were no significant differences between the different concentrations. The proportion of GFAP-positive astrocyte-like cells in the different concentrations of ginkgolide B component was not only higher than that in the control group, but also increased with ginkgolide B concentration. However, different concentrations of ginkgolide B had little effect on the differentiation of glial-like cells into oligo4-positive oligodendrocytes [47,61].

**Table 3:** The role of Chinese herbs and its active ingredients to promote bone marrow mesenchymal stem cells differentiate into nerve cells

**Chinese herbs and active ingraients**	**Types of MSCs**	**The role of proliferation**	**The role of differentiation**	**Mechanism**
*Salvia*[62-64]	MSCs of mouse	Nestin cells↑	β-tubulin cells↑	
	MSCs of rat	Nestin cells↑	NSE cells↑	Expression of Ngn-1 and Mash-1↑
	MSCs of human	Nestin cells↑	NF cells↑	
*Astragalus*[65]	MSCs of rat		NSE cells↑	Expression of Ngn-1 and Mash-1↑
Ginsenoside Rg1 [66,67]	MSCs of rat		NSE cells↑	Expression of NGFmRNA↑
*P. notoginseng* saponins + retinoic acid [33-37]	MSCs of rat		NSE cells↑ DA, GABA cells	
Musk polypeptide, Muscone [43]	MSCs of rat		T NSE and NF cells↑	
Ginkgolide B [47,60,61]	MSCs of rat		NSE cells ↑ (Has nothing to do with the concentration). The number of GFAP cells GFAP↑ (Positively correlated with the concentration).	

Five hours after *Salvia* (*Dan Shen*) induction, most rat MSCs expressed NSE, and NF-positive brownish yellow staining, morphological diversity, simple bipolar cells and complex multi-polar cells [62]. GFAP immunohistochemistry was negative. While the control group did not have any nestin positive cells, the majority of cells showed nestin positivity by 5 h after *Salvia* induction, indicating that the cells had neural stem cell characteristics. The results of reverse-transcription polymerase chain reaction showed that *mash-1* and *ngn-1* were not expressed in MSCs before induction, but they were expressed after induction. *Salvia* induced differentiation of neuron-like cells in addition to the morphological characteristics of neurons at the genetic level [63,64].

There were no cell morphological changes in rat MSCs by 24 h after injection of *Astragalus* (20 μL/mL), but visible morphological changes in a small number of cells by 24 h after injection of *Astragalus* (50 μL/mL) [65]. Some cell morphology changes were observed in some cells only 5 h after injection of *Astragalus* (100 μL/mL). Up to 24 h after the injection, more neuron-like cells, the formation of slender processes, and network-like connections between the visible parts of cells were observed. Nestin-positive cell staining was strongest 24 h after injection of *Astragalus* (100 μL/mL). The numbers of NSE- and GFAP-positive cells were highest 24 h after injection of *Astragalus* (200 μL/mL). Only a small number of stained MAP-2-positive cells were observed 24 h after injection of *Astragalus* (100 and 200 μL/mL). The expression levels of *Wnt-1* and *Ngn*-1 genes, which play a positive regulatory role in the differentiation process, were significantly higher [65,66]. MSCs of rat cultured with serum-free ginseng saponin Rgl (10 μmol/L) for 3 days, some cells expressed NSE, but GFAP staining was negative [66]. The level of NGF mRNA in the ginsenoside Rgl-treated group was significantly higher than that in the control group, suggesting that ginsenoside Rgl induced neuron-like cells to express NGF mRNA [67].

### Limitations and further studies

Current research is limited to the effects of a selection of Chinese herbs and their active components on neural stem cell proliferation and differentiation. The mechanisms of their actions are largely unknown. This review is based on animal studies.

## Conclusion

Specific Chinese herbs exhibited effects on the differentiation, proliferation and activation of neural stem cells and mesenchymal stem cells.

## Abbreviations

CM: Chinese medicine; SVZ: Subependymal zone; SGZ: Subgranular zone; AD: Alzheimer’s disease; NSCs: Neural stem cells; DG: Dentate gyrus; MSCs: Mesenchymal stem cells; NSE: Neuron-specific enolase; RRTP: Rose roxburghii tratt polysaccharide; NT-3: Neurotrophin-3; NF: Neurofilament proteins; NGF: Nerve growth factor.

## Competing interests

All authors declare that they have no competing interests.

## Authors’ contributions

YS, QL, CX, and XN conducted this review. YS, QL, XX and CY wrote the manuscript. All the authors read and approved the final version of the manuscript.
